# Three-Dimensional Culture of Cartilage Tissue on Nanogel-Cross-Linked Porous Freeze-Dried Gel Scaffold for Regenerative Cartilage Therapy: A Vibrational Spectroscopy Evaluation

**DOI:** 10.3390/ijms23158099

**Published:** 2022-07-22

**Authors:** Tetsuya Adachi, Nao Miyamoto, Hayata Imamura, Toshiro Yamamoto, Elia Marin, Wenliang Zhu, Miyuki Kobara, Yoshihiro Sowa, Yoshiro Tahara, Narisato Kanamura, Kazunari Akiyoshi, Osam Mazda, Ichiro Nishimura, Giuseppe Pezzotti

**Affiliations:** 1 Department of Dental Medicine, Graduate School of Medical Science, Kyoto Prefectural University of Medicine, Kamigyo-ku, Kyoto 602-8566, Japan; n-miya@koto.kpu-m.ac.jp (N.M.); hyt8888@outlook.jp (H.I.); yamamoto@koto.kpu-m.ac.jp (T.Y.); elia-marin@kit.ac.jp (E.M.); kanamura@koto.kpu-m.ac.jp (N.K.); 2 Department of Immunology, Graduate School of Medical Science, Kyoto Prefectural University of Medicine, 465 Kajii-cho, Kamigyo-ku, Kyoto 602-8566, Japan; sowawan@koto.kpu-m.ac.jp (Y.S.); mazda@koto.kpu-m.ac.jp (O.M.); 3 Infectious Diseases, Kyoto Prefectural University of Medicine, 465 Kajii-cho, Kamigyo-ku, Kyoto 602-8566, Japan; 4 Ceramic Physics Laboratory, Kyoto Institute of Technology, Sakyo-ku, Matsugasaki, Kyoto 606-8585, Japan; wlzhu@kit.ac.jp; 5 Department of Clinical Pharmacology, Division of Pathological Science, Kyoto Pharmaceutical University, Misasagi Nakauchi-cho, Yamashina-ku, Kyoto 607-8414, Japan; kobara@mb.kyoto-phu.ac.jp; 6 Department of Plastic and Reconstructive Surgery, Kyoto Prefectural University of Medicine, 465 Kajii-cho, Kamigyo-ku, Kyoto 602-8566, Japan; 7 Department of Plastic and Reconstructive Surgery, Graduate School of Medicine, Kyoto University, Yoshida-Konoe-cho, Sakyo-ku, Kyoto 606-8501, Japan; 8 Department of Chemical Engineering and Materials Science, Doshisha University, 1-3 Tatara Miyakodani, Kyotanabe, Kyoto 610-0394, Japan; ytahara@mail.doshisha.ac.jp; 9 Department of Polymer Chemistry, Graduate School of Engineering, Kyoto University, Katsura, Nishikyo-ku, Kyoto 615-8510, Japan; akiyoshi@bio.polym.kyoto-u.ac.jp; 10 Division of Oral Biology and Medicine, The Jane and Jerry Weintraub Center for Reconstructive Biotechnology, UCLA School of Dentistry, Los Angeles, CA 90095, USA; inishimura@dentistry.ucla.edu; 11 Division of Advanced Prosthodontics, The Jane and Jerry Weintraub Center for Reconstructive Biotechnology, UCLA School of Dentistry, Los Angeles, CA 90095, USA; 12 Biomedical Research Center, Kyoto Institute of Technology, Sakyo-ku, Matsugasaki, Kyoto 606-8585, Japan

**Keywords:** NanoCliP-FD gel scaffold, tissue engineering, regenerative therapy, human periodontal ligament-derived stem cell, in situ Raman spectroscopy

## Abstract

This study presents a set of vibrational characterizations on a nanogel-cross-linked porous freeze-dried gel (NanoCliP-FD gel) scaffold for tissue engineering and regenerative therapy. This scaffold is designed for the in vitro culture of high-quality cartilage tissue to be then transplanted in vivo to enable recovery from congenital malformations in the maxillofacial area or crippling jaw disease. The three-dimensional scaffold for in-plate culture is designed with interface chemistry capable of stimulating cartilage formation and maintaining its structure through counteracting the dedifferentiation of mesenchymal stem cells (MSCs) during the formation of cartilage tissue. The developed interface chemistry enabled high efficiency in both growth rate and tissue quality, thus satisfying the requirements of large volumes, high matrix quality, and superior mechanical properties needed in cartilage transplants. We characterized the cartilage tissue in vitro grown on a NanoCliP-FD gel scaffold by human periodontal ligament-derived stem cells (a type of MSC) with cartilage grown by the same cells and under the same conditions on a conventional (porous) atelocollagen scaffold. The cartilage tissues produced by the MSCs on different scaffolds were comparatively evaluated by immunohistochemical and spectroscopic analyses. Cartilage differentiation occurred at a higher rate when MSCs were cultured on the NanoCliP-FD gel scaffold compared to the atelocollagen scaffold, and produced a tissue richer in cartilage matrix. In situ spectroscopic analyses revealed the cell/scaffold interactive mechanisms by which the NanoCliP-FD gel scaffold stimulated such increased efficiency in cartilage matrix formation. In addition to demonstrating the high potential of human periodontal ligament-derived stem cell cultures on NanoCliP-FD gel scaffolds in regenerative cartilage therapy, the present study also highlights the novelty of Raman spectroscopy as a non-destructive method for the concurrent evaluation of matrix quality and cell metabolic response. In situ Raman analyses on living cells unveiled for the first time the underlying physiological mechanisms behind such improved chondrocyte performance.

## 1. Introduction

When attempted with surgical treatments, the regeneration rate of articular cartilage is generally inherently low and might result in the formation of a fibrous tissue inferior to native cartilage [1]. Accordingly, new strategies involving the use of chondrocytes have been developed, in attempts to increase the regeneration rate while also improving the quality of cartilage tissue [2]. In options that utilize implanted cells, limitations arise from the low number of cells available for isolation. However, attempts to expand in vitro the chondrocyte cell cultures usually lead to the occurrence of undesirable changes in cell phenotype [3]. In this latter context, the use of articular chondrocytes and mesenchymal stem cells (MSCs) in coculture has been found to stimulate chondrocyte redifferentiation, thus enhancing the possibility to attain chondrocyte phenotype stability in vitro [4].

Recent advances in tissue engineering and regenerative treatments have enabled the transplantation of cartilage tissues cultured in vitro into patients with congenital malformations of the maxillofacial region or jaw disease [5]. However, while the differentiation of MSCs into cartilage tissue usually takes place in culture, the simultaneous process of dedifferentiation is also observed, which makes it difficult to construct cartilage tissue of sufficient size and quality [6]. Dedifferentiation of chondrocytes, which is defined as the gradual loss of molecular markers defining differentiated chondrocytes, yet represents the main shortcoming in culturing cartilage.

As for the currently available methods to evaluate cultured cartilage tissues, to date, traditional methods have relied on destructive tests of cartilage tissue, which screen gene expression and substrate production by reverse transcription–polymerase chain reaction (RT–PCR) and immunostaining, respectively. There are as yet no available in situ procedures to non-destructively evaluate the “differentiation status” of living cells and the quality of cartilage tissue they produce. Moreover, as a large amount of sample is required for the above conventional analyses, their destructive nature creates cost problems as well as a serious burden for the donor. For the aforementioned reasons, we aimed to develop Raman and infrared spectroscopic procedures that allow comprehensive analyses at the molecular level in a non-destructive and non-invasive manner. Using Raman spectroscopy, we developed a technology for the real-time monitoring of the differentiation state of regenerated cartilage tissue and of the “quality” of the cartilage matrix at the molecular level, including information on its steric construction. The data presented in this study build upon previous studies of cross-linked pullulan polysaccharide-based nanogels and the subsequent development of a nanogel-cross-linked porous freeze-dried (NanoCliP-FD) gel scaffold [7,8,9,10,11]. As a supplementary output, this study completes our previous Raman spectroscopic characterizations of human cartilage tissue explanted from both healthy and osteoarthritic patients and osteoarthritic cartilage regeneration by mRNA therapeutics [12,13,14]. The proposed spectroscopic procedures could supplement and complete the reports on cell culture features, or be used as a preliminary step, prior to PCR and immunostaining.

In this study, we succeeded in stimulating MSCs to construct cartilage tissue at a rate more than fivefold faster than that obtained from MSCs cultured on conventional scaffolds by employing a NanoCliP-FD gel scaffold. The aim of the present study was to construct at a high rate relatively large portions of high-quality cartilage tissue by three-dimensionally culturing MSCs on biocompatible scaffolds. Concurrently, we developed a technology to evaluate the cartilage matrix quality and the metabolic response of chondrocytes in a non-destructive manner, based on a Raman spectroscopic approach. We anticipate that the procedures presented in this study could be utilized in clinical applications as a new method for novel cartilage regeneration treatment while monitoring the quality of regenerative cartilage in situ and non-destructively through Raman spectroscopic analyses.

## 2. Results

### 2.1. Laser Microscopy Observation and Immunohistochemical Analyses

**Figure 1:**
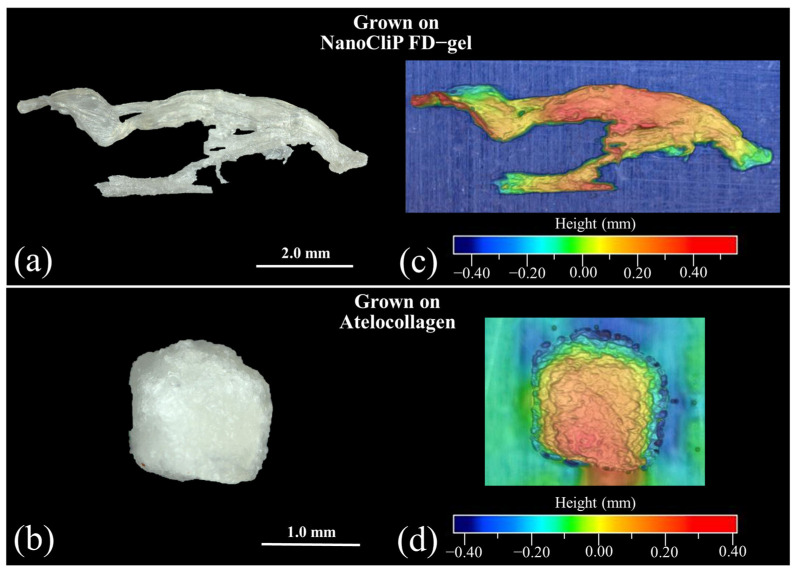
Laser microscope images of cartilage tissue formed after culturing for 14 days onto (**a**) NanoCliP-FD and (**b**) atelocollagen scaffolds; (**c**,**d**) represent their respective 3D views.

PDLSCs, a type of MSC, are known to differentiate into a variety of cells, including osteoblasts, cartilage cells, adipose cells, and nerve cells [15,16,17,18]. Choi et al. [19] first reported the potential of PDLSCs as a valuable cell source for chondrogenesis. PDLSCs may be applicable to the regeneration and reconstruction of cartilage in the field of cell therapy [19]. Cartilage tissue morphology was observed after 14 days using laser microscopy. Figure 1a,b show pictures of the cartilage tissue grown onto NanoCliP-FD gel and atelocollagen scaffolds, respectively, and Figure 1c,d represent their respective three-dimensional views. Weight measurements on the scaffolds revealed that cartilage tissue grew at a speed ~5 times faster on the NanoCliP-FD gel scaffold compared to the atelocollagen one.

**Figure 2:**
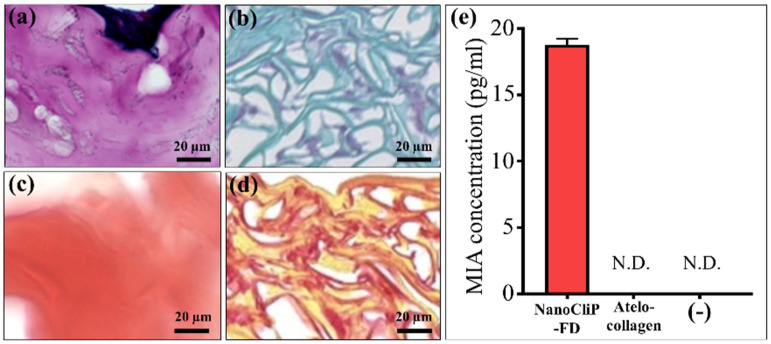
Light microscopy images of Safranin O staining in chondrogenic medium ((**a**,**b**) for NanoCliP-FD gel and atelocollagen, respectively) and of Picrosirius red staining ((**c**,**d**) for NanoCliP-FD gel and atelocollagen, respectively). In (**e**), the results of MIA concentrations in culture supernatant at 14 days culture are compared between NanoCliP-FD gel and atelocollagen scaffolds. The (-) symbol represents the negative control (no scaffold).

The cell-grown cartilage matrix was then stained using Safranin O stain and Picrosirius red stain in order to perform a histological evaluation. In a cartilage-inducing medium, NanoCliP-FD gel (Figure 2a,c for Safranin O and Picrosirius Red, respectively) had a deeper reddish-purple hue and a greater amount of cartilage matrix compared to atelocollagen (Figure 2b,d for Safranin O and Picrosirius red, respectively). In normal medium-based cultures, no dark stains were observed in either atelocollagen or NanoCliP-FD gel (data not shown). In an additional evaluation, melanoma MIA levels were measured in the supernatant using the ELISA kit. MIA is a cartilage-derived retinoic acid-sensitive protein (CD-RAP) and it represents a differentiation marker for cartilage [9]. ELISA results showed that only the cartilage tissue on NanoCliP-FD gel produced MIA in the culture supernatant at 14 days culture (Figure 2e).

**Figure 3:**
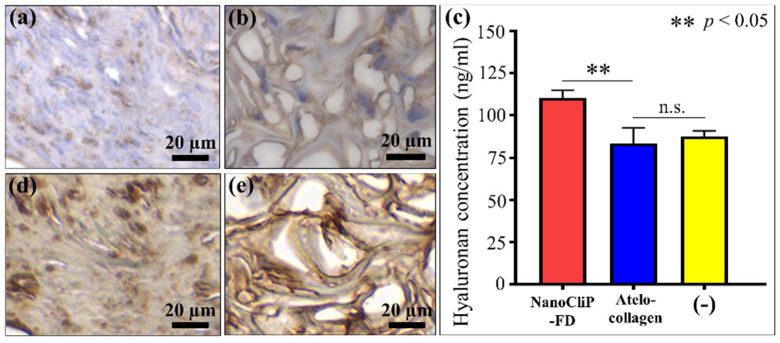
Light microscopy images of biotinylated hyaluronan-binding protein staining of cartilage tissue grown in CM onto (**a**) NanoCliP-FD gel and (**b**) atelocollagen scaffolds; (**c**) ELISA measurements to determine the soluble hyaluronan supernatant concentration at 14 days of culture (statistical results of Student’s *t-*test showed significance between data collected on different scaffolds; the abbreviation n.s. means non-significant); (**d**,**e**) show light microscopy images are shown of stained type II collagen in the cartilage tissue grown in CM onto NanoCliP-FD gel and atelocollagen, respectively.

Histological evaluations of hyaluronan expression within the cartilage matrix were performed using a VeriScan of specific hyaluronan-bonded biotin labels. Consistent with data in Figure 2, these analyses showed that the cartilage tissue produced by cells onto the NanoCliP-FD gel scaffold (Figure 3a) was more abundant in hyaluronan than that formed of the atelocollagen-based structure (Figure 3b). As a further confirmation, hyaluronan production was also measured in the supernatant. Hyaluronan, a type of straight-chain glycosaminoglycan (mucopolysaccharide), non-covalently bonds with aggrecan and links proteins. The ultra-high molecular weight complex formed by these bonds plays a significant role in the maintenance of cartilage function. ELISA results showed that the cartilage-induced NanoCliP-FD gel produced the highest amount of hyaluronan in culture supernatant (Figure 3c). Finally, we also conducted a histological evaluation of type II collagen expression within the cartilage matrix by means of immunostaining. The type II collagen in the cartilage tissue that formed onto the NanoCliP-FD gel scaffold (Figure 3d) showed no significant difference in stain intensity compared with that formed on the atelocollagen-based structure (Figure 3e).

In summary, the complete set of immunohistochemical data confirmed that three-dimensional culturing of MSCs is more efficient on the NanoCliP-FD gel scaffold and results in higher cell differentiation compared to the atelocollagen scaffold with no significant difference in the amount of type II collagen.

### 2.2. Scaffolds and Their Raman Spectroscopic Evaluations

Extensive characterizations of the two selected scaffolds were described in a previous paper [10]. Briefly, both scaffolds presented highly wrinkled and porous morphologies. The fibrous NanoCliP-FD gel scaffold possessed a hierarchical structure with a bimodal pore distribution including macro-sized (interconnected) pores of several hundred micrometers and a fine porosity associated with nano-domains (arising from phase separation upon gelation) [7]. In contrast, the spongy atelocollagen scaffold, which is conventionally used in tissue-regenerative procedures, possessed a high degree of large porosity and pore interconnectivity that facilitates initial cell impregnation, successive cell-to-cell interactions, mass transfer of nutrients, and promotion of tissue ingrowth [20].

**Figure 4:**
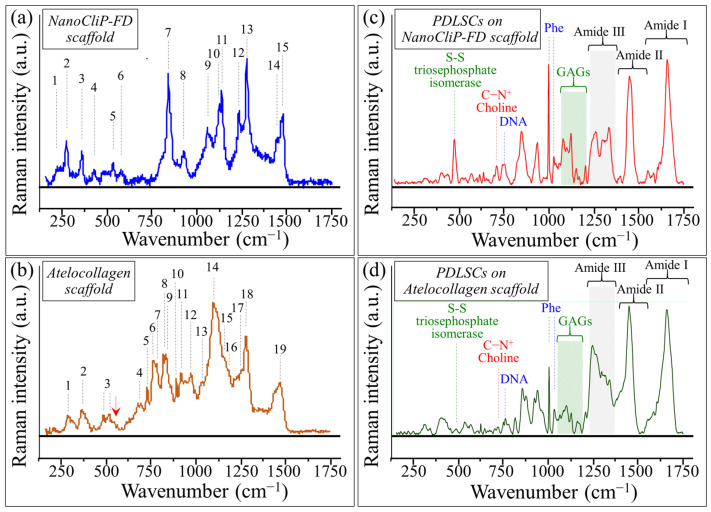
Average Raman spectra collected on (**a**) NanoCliP-FD gel and (**b**) atelocollagen scaffolds before exposure to PDLSCs; the labels of the main spectroscopic bands correspond to those listed in Table 1 and Table 2, respectively. The red arrow in (**b**) represents a characteristic signal from α-helical polypeptide poly (γ-benzyl glutamate). In (**c**,**d**), Raman spectra are shown of cartilage tissue grown by PDLSCs on NanoCliP-FD gel and atelocollagen scaffolds, respectively. Both spectra are normalized to the amide I band intensity (labels are explained in the text).

Average Raman spectra collected on both NanoCliP-FD gel and atelocollagen scaffolds before exposure to PDLSCs are shown in Figure 4a,b, respectively. The main bands in both spectra were labeled and their vibrational origins assigned as shown in Table 1 and Table 2, respectively. The NanoCliP-FD gel scaffold is basically a pullulan structure, namely, a long-chain structure belonging to the family of α-D-glucose polymers. Accordingly, the spectral region in the wavenumber interval 200 to 600 cm^−1^, which comprises Bands 1 to 4, can be assigned to C-O torsional and skeletal vibrations in glucose [21]. Bands 5 and 6 (at 534 and 580 cm^−1^, respectively) represented glucose C-O stretching, although the former band might also include contributions from glucose C-C stretching [22]. In contrast, Bands 7 and 8 (at 843 and 921 cm^−1^, respectively) can be assigned to glucose anomeric C-H bending and C-O-C bending vibrations, respectively [23]. C-C-H bending in glucose (Band 9) and C-O-C stretching signals from ester groups (Band 10) clearly appeared at 1060 and 1125 cm^−1^, respectively. At higher frequencies, the amide III band displayed at ~1234 cm^−1^ (Band 11), and the triplet composed by Bands 13 to 15 in the wavenumber interval 1270 to 1480 cm^−1^ is mainly related to CH or CH_2_ bending modes. The amide III band at 1234 cm^−1^ (Band 12) can be taken as a fingerprint for amination [24], namely the graft of amine groups to the polysaccharide chain. Moreover, the presence of the C-O-C stretching signal at 1125 cm^−1^ can be attributed to the occurrence of the esterification reaction [24]. C-O-C stretching in esters is also expected to give a signal at ~1267 cm^−1^ in partial overlap with Band 13.

The Raman spectrum of the atelocollagen scaffold is given in Figure 4b and its labeled bands are listed in Table 2. Vibrational assignments were made according to previously published literature [25,26,27,28,29]. The low-frequency Raman interval (200–600 cm^−1^) mainly represented the vibrational modes of polypeptides and proteins. The torsional modes of the polypeptide backbone usually include three specific compounds: α-helical polypeptides, the triple-helical polymer (Pro-Pro-Gly)_10_, and protein collagen. However, the triple-helical (Pro-Pro-Gly)_10_ polymeric structure displayed specific spectroscopic fingerprints at 307 and 402 cm^−1^ (Bands 1 and 2, respectively). The doublet recorded at 561 and 763 cm^−1^ (Bands 3 and 4, respectively), which is referred to as the amide VI band, mainly occurs in dipeptides and is associated with the presence of the –CO–NH– sub-structure [25]. It is of note that the spectrum of the α-helical polypeptide poly (γ-benzyl glutamate) was silent in the 300 to 550 cm^−1^ region, but exhibited a doublet at 554 and 576 cm^-l^ and an additional band at 611 cm^−1^. The former two Raman signals were likely embedded in the broad amide VI signal (at around Band 3), while the band at 611 cm^−1^ (labeled with a red arrow in Figure 4b) appeared as a high-frequency shoulder. The sharp signal detected at around 816 cm^−1^ (Band 5), which represented C-O-C stretching vibrations, has been associated with glucosyl-galactosyl-hydroxylysinonorleucine cross-links between tropocollagens [25]. The bands located at 858, 874, and 921 cm^−1^ (Bands 6–8) arose from C-C vibrations in the hydroxyproline ring, while the signal at 938 cm^−1^ (Band 9) corresponded to the C–C stretching vibration of the α-helix backbone [26]. The two signals at 1004 and 1034 cm^−1^ (Bands 10 and 11, respectively) belong to C-C stretching in phenylalanine [26] and in-plane C-OH bending [27,28], respectively. Band 12 (at 1106 cm^−1^) is difficult to assign with certainty; it could represent C-N stretching [27] or be interpreted as a signal for CH_2_ wagging mode in glutamic acid, the only amino acid residue that exhibits it [28]. It is of note that Band 12 was quite pronounced in the spectrum of the atelocollagen scaffold, which might suggest the presence of a conspicuous fraction of glutamic acid in this scaffold structure. Band 13 (at 1177 cm^−1^) was observed as a relatively weak low-frequency shoulder to the amide III Band 14. This band is a fingerprint for tyrosine residue and is related to vibrational in-plane C-C-H bending vibrations of the phenol ring [29]. Bands 14 to 16 (at 1247, 1268, and 1324 cm^−1^, respectively) represent amide III vibrations, whereas Band 17 (at 1420 cm^−1^) arose from the COO^-^ stretching mode [26]. Band 18 (at 1455 cm^−1^) represented CH_2_ and CH_3_ bending modes, and Band 19, which was recorded at 1655 cm^−1^, is the amide I vibration.

Understanding the vibrational origins of the Raman bands recorded in the two different scaffolds and their variations upon exposure to cells (see next Section 2.3) helps separate bands from scaffolds and cells, and allows analysis of cell/scaffold interactions, which, in turn, enables interpretations of the differences in chondrogenic behavior revealed by the immunohistochemical analyses presented in Section 2.1.

### 2.3. Raman Spectroscopic Evaluations of PDLSC-Grown Cartilage Tissues

Raman spectra of cartilage tissue grown by PDLSCs on NanoCliP-FD gel and atelocollagen scaffolds (normalized to the amide I intensity) are shown in Figure 4c,d, respectively. In the low-frequency (250~500 cm^−1^) zone of the Raman spectrum of cartilage tissue grown on the NanoCliP-FD gel scaffold, the almost complete disappearance of the scaffold-related Bands 2 and 3 (C-O torsion at 276 cm^−1^ and C-C-O bending at 359 cm^−1^, respectively) was observed, as well as a significant reduction in all other neighboring bands related to glucose C-O bonds in the same spectral region. In addition, a main feature appeared with a new sharp band located at around 495 cm^−1^. In the cartilage tissue grown on the NanoCliP-FD gel scaffold, the band was in a peculiar position for S-S stretching, which can be assigned to triosephosphate isomerase (TPI) [30] (cf. label in Figure 4c). TPI is an extremely efficient enzyme that catalyzes the interconversion of two triose phosphate isomers: dihydroxyacetone phosphate (DHAP) and (R)-glyceraldehyde-3-phosphate (G3P). The catalytic function of TPI is essential in the glycolytic pathway, a complex 10-step cytoplasmic reaction that takes place in cartilage metabolism [31,32]. Ubiquitous in mammalians, TPI carries out an essential role in the glycolytic pathway by which one glucose molecule converts into two molecules of 3-carbon pyruvate, in addition to generating two molecules of adenosine triphosphate (ATP) and transforming the oxyreductive coenzyme nicotinamide adenine dinucleotide (NAD+) into its reduced form NADH [32]. These latter two molecules act as metabolic fuels in driving a number of biological reactions [33] and are known to regulate energy metabolism in chondrocytes [34]. It is of note that the band at 495 cm^−1^ was very weak in the cartilage tissue grown onto the atelocollagen scaffold (cf. Figure 4d). This result should be interpreted as a boosting effect of TPI enzymatic reactions that only occur in chondrocytes cultured on NanoCliP-FD gel, which is rich in glucose molecules (i.e., the basic molecule that aliments TPI catalytic reactions). An additional argument in favor of an increased functionality of chondrocytes cultured on the NanoCliP-FD gel scaffold resides in the conspicuous disappearance of the torsional and bending modes of C-O bonds in the glucose rings of the pullulan structure (cf. above), which suggests a process of the gradual consumption of the pullulan structure (through TPI catalytic activity) concurrent with the formation of new cartilage tissue.

As a common feature, both spectra in Figure 4c,d displayed the sharp signal of phenylalanine (Phe) at ~1001 cm^−1^ (and its companion one at 1034 cm^−1^) from benzene ring vibrations [35] (cf. labels). However, the relative intensity of the band at 1001 cm^−1^ was higher in the tissue grown on the NanoCliP-FD gel scaffold compared to the atelocollagen one; this result might be interpreted as an increased cell population on the former scaffold under the same culture conditions. There are two pieces of additional evidence in support of this thesis: (i) the higher signal intensity of the C-N^+^ stretching band at ~720 cm^−1^ as observed in tissue grown onto the NanoCliP-FD gel scaffold; this band belongs to the quaternary ammonium salt choline unit (cf. labels in Figure 4c,d) and is a fingerprint for lipids in the chondrocytes’ membrane [36]; and, (ii) the consistently higher signal intensity of the chondrocytes’ DNA band at ~750 cm^−1^ (ring breathing).

**Figure 5:**
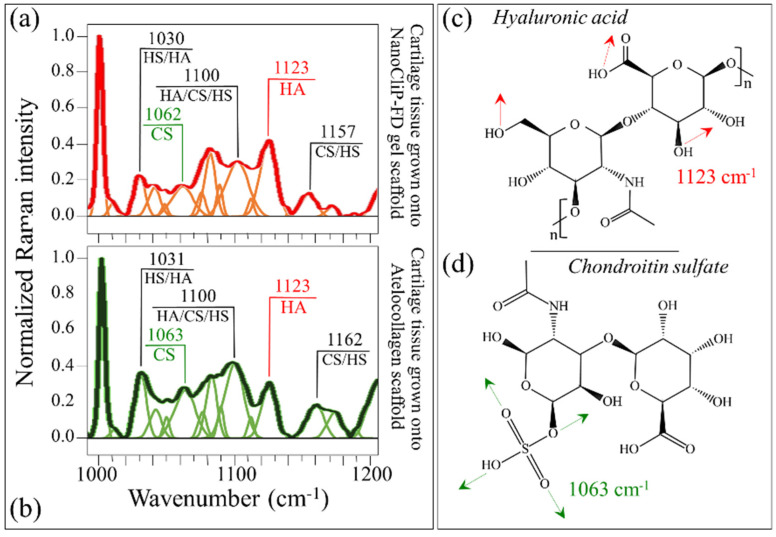
The Raman spectral zone between 1000 and 1200 cm^−1^ (characteristic of GAGs) is deconvoluted into sub-bands related to CS, HA, and HS (cf. labels): cartilage grown onto (**a**) NanoCliP-FD gel and (**b**) atelocollagen scaffolds. Structure and fingerprint bands for HA (bending vibrations of C-OH groups) and CS (O-S-O_3_^–^ symmetric stretching) are given in (**c**,**d**), respectively.

The Raman spectral zone between 1000 and 1200 cm^−1^ predominantly displayed signals from glycosaminoglycans (GAGs) contained in the cartilage tissue. Several bands, which are contributed by chondroitin sulfate (CS), hyaluronic acid (HA), and heparan sulfate (HS), could similarly be deconvoluted in the NanoCliP-FD gel and atelocollagen scaffolds although with different relative intensities (Figure 5a,b, respectively). The peak at ~1123 cm^−1^, which is related to the bending vibrations of C-OH groups [37], was unique to HA (Figure 5c). Conversely, the band at ~1063 cm^−1^, which arose from O-S-O_3_^–^ symmetric stretching, was a fingerprint for CS [38] (Figure 5d). Two additional bands at 1030 and 1100 cm^−1^ represented overlapping contributions from C-C and C-O stretching vibrations, and C-OH bending vibrations of acetyl groups, respectively [39,40]. These signals were cumulatively built from several GAGs’ contributions and cannot selectively locate any specific GAG. The band at 1160 cm^−1^ also contained composite contributions since it arose from sugar ring moieties in both GAGs and proteoglycans; more specifically, it reflected vibrations of exocyclic (–C–O–C–) inter-molecular groups [40]. There are important implications of the marked differences in relative intensities in the GAGs spectral zone between cartilage tissue grown onto different scaffolds. An important feature was the relative intensity between the CS peak at 1063 cm^−1^ and the HA peak at 1123 cm^−1^. The latter band was significantly stronger than the former in cartilage tissue grown onto the NanoCliP-FD gel scaffold (cf. Figure 5a). A different trend was observed for the case of the atelocollagen scaffold, in which the CS and HA bands presented comparable intensities (cf. Figure 5b). Kamilari et al. [41] proposed using the intensity ratio of the deconvoluted bands at 1123 and 1063 cm^−1^ (i.e., the ratio *I*_1123_/*I*_1063_; as fingerprints of HA and CS, respectively) to quantify GAG species by Raman spectroscopy. The ratio *I*_1123_/*I*_1063_ was computed as 2.45 and 1.11 for NanoCliP-FD gel and atelocollagen scaffolds, respectively. According to their calibration experiments, the content of HA in the cartilage tissue grown onto the NanoCliP-FD gel scaffold was nearly twofold that of CS, whereas HA and CS contents were comparable in the case of the atelocollagen scaffold. The physiological meaning of this structural difference will be discussed further in the forthcoming Section 3.

**Figure 6:**
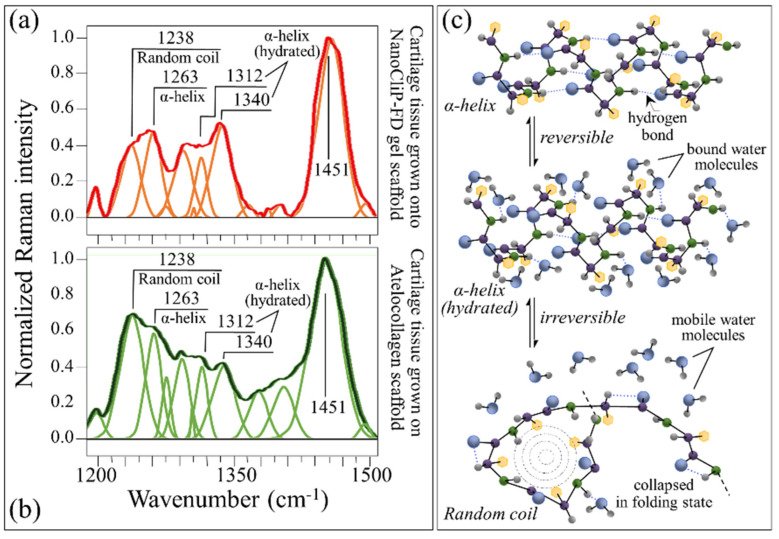
Deconvoluted spectra in the amide III wavenumber region are shown for cartilage tissue grown onto (**a**) NanoCliP-FD gel and (**b**) atelocollagen scaffolds. In (**c**), schematic drafts are given for α-helix, its reversible hydrated configuration, and its irreversibly disordered random coil structures (from top to bottom).

The amide III spectral zone in the wavenumber interval 1170 to 1500 cm^−1^ (cf. labels in Figure 4c,d) provides structural information on the collagen structure of cartilage tissue. Deconvoluted spectra in the amide III wavenumber region are shown in Figure 6a,b for tissues grown onto NanoCliP-FD gel and atelocollagen scaffolds, respectively. Deconvolution of this zone into a series of sub-bands (cf. labels) revealed significant differences. The strongest band in this region, which appeared at around 1451 cm^−1^, was a composite signal of two largely overlapping sub-bands assigned to methyl CH_3_ and methylene CH_2_ deformations [26]. This cumulative band was used for normalization in this spectral zone (cf. Figure 6). One important feature was the observed difference in relative intensity ratio between the amide III bands at 1238 cm^−1^ and 1263 cm^−1^ (cf. labels in Figure 6). Despite having the same physical origin (N-H in-plane bending), these bands displayed at clearly shifted wavenumbers because they represented different protein secondary structures, namely, the random coil (disordered) and the α-helix (ordered) structures, respectively [42]. It is important to note that the bands at 1451 and 1238 cm^−1^ did not show orientation dependence in polarized probe assessments and thus exploited homogeneous Raman scatter [43]. Therefore, the relative intensity of the 1238 cm^−1^ to the 1451 cm^−1^ band can be assumed to be a measure of the fractional amount of random coil in the cartilage collagen structure. Accordingly, the intensity ratios *I*_1263_/*I*_1451_ represents a spectroscopic parameter linked to the degree of disorder of the collagen structure. According to this approach, the protein structure in the cartilage collagen grown onto the atelocollagen scaffold was found to possess a random coil fraction 32% higher and, thus, a more disordered (defective) structure, compared to the collagen tissue developed in the NanoCliP-FD gel scaffold under the same culture conditions.

Another important feature in the amide III region was represented by the two signals at ~1312 and ~1340 cm^−1^, both of which belonged to α-helix in its hydrated state [44,45,46,47]. The hydrated variants of α-helix possess an open conformation, which leaves the hydrogen-bonding network intact but changes the C-O⋯N angle in the amide plane to allow carbonyls being displaced away from the helix axis into the solution and hydrogens bonding with environmental water molecules. The ratio of the cumulative intensity of the two bands at ~1312 and ~1340 cm^−1^ to that of the α-helix band at 1263 cm^−1^, *I*_1312+1340_/*I*_1263_, could be taken as a parameter representative of the degree of cartilage hydration. Such computation shows a value of the ratio for cartilage grown on NanoCliP-FD gel scaffold approximately 27% higher than that of cartilage on the atelocollagen scaffold (1.79 vs. 1.41). Figure 6c shows schematic drafts of α-helix, its hydrated configuration, and the random coil structures. Note that the observed higher level of tissue hydration in the NanoCliP-FD gel scaffold was consistent with its higher HA amount [48], as detected in the GAGs spectral region (cf. above in this section). The presence of a highly hydrated structure was only characteristic of the very surface zone of native cartilage, whereas a lower collagen-to-GAG ratio, and the associated increase in tissue hydration, is a common bulk characteristic of engineered cartilages compared to native tissue. The hydrophilic properties of proteoglycans exposed at the surface of articular cartilage enhance tissue hydration and favor low-friction sliding characteristics [49,50].

### 2.4. Raman Imaging of Cartilage Tissue Grown onto Different Scaffolds

**Figure 7:**
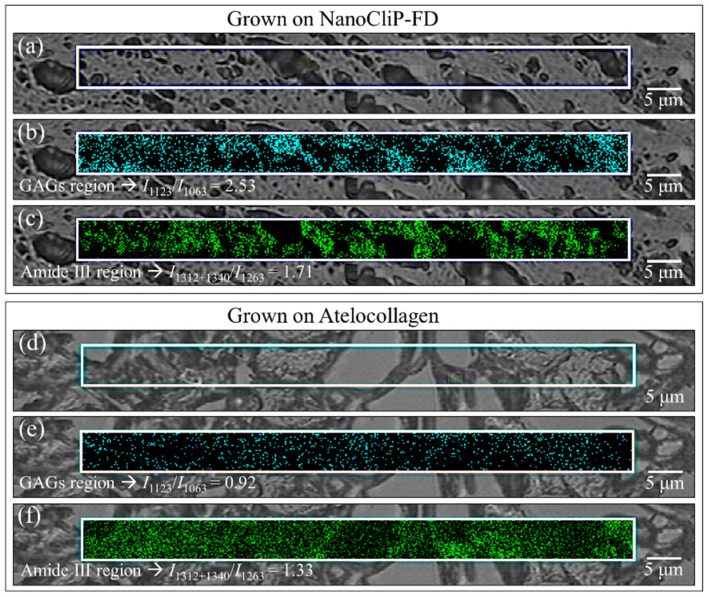
Raman maps collected on cartilage tissue grown onto NanoCliP-FD gel (**a**,**c**) and atelocollagen (**d**–**f**) scaffolds: (**a**,**d**) are optical micrographs, (**b**,**e**) represent the spatial distribution of the ratio *I*_1123_/*I*_1063_ (measuring the fractional ratio of HA to CS), (**c**,**f**) show the spatial distribution of the ratio *I*_1312+1340_/*I*_1263_ (measuring the degree of α-helix hydration).

The data presented in the previous sections highlighted the fundamental differences in both structure and functionality between Raman spectra collected on cartilage tissue grown onto NanoCliP-FD gel and atelocollagen scaffolds under exactly the same culture conditions. The detected differences included GAGs fractions and protein secondary structures in collagen, which were represented by the spectroscopic ratios *I*_1123_/*I*_1063_, as a fingerprint of the fractional ratio of HA to CS, and *I*_1312+1340_/*I*_1263_, as a measure of the degree of α-helix hydration in the collagen structure. In order to gain statistical accuracy and to confirm spectroscopic findings on average spectra (in Section 2.3), Raman mapping was performed with micrometric spatial resolution in order to image compositional differences in cartilage tissue grown on different scaffolds. Figure 7 shows optical images and Raman maps collected on cartilage tissue grown onto NanoCliP-FD gel (a–c) and atelocollagen (d–f) scaffolds. Each map covers ~500 μm^2^ and is composed of 1.2 × 10^4^ spectra collected with a lateral step of 0.2 μm. The Raman maps in (b) and (e) (taken in correspondence with the locations shown in micrographs (a) and (d), respectively) represent the spatial distribution of the ratio *I*_1123_/*I*_1063_ (measuring the fractional ratio of HA to CS). In contrast, the maps in (c) and (f) (corresponding to the locations in (a) and (d), respectively) show the spatial distribution of the ratio *I*_1312+1340_/*I*_1263_ (measuring the degree of α-helix hydration). The “mapping approach” could be considered as complementary to the “average approach” followed in the previous section, in which representative (average) spectra were obtained upon averaging thirty spectra collected at different locations with a relatively low spatial resolution (20× optical lens) but covering relatively large areas of the scaffolds (in the order of mm^2^) with high spectral resolution. Average values for the two selected Raman biochemical parameters are given in the inset to each section of Figure 7. Raman mapping analyses confirmed that the cartilage tissue grown onto the NanoCliP-FD gel scaffold was richer in HA and its collagen had a more hydrated protein α-helix structure than that grown under exactly the same conditions on the atelocollagen scaffold. Remarkably, the results of the two complementary Raman approaches (i.e., “mapping” and “average”) gave consistent results to each other with differences of <10%. This result can be interpreted as a confirmation of the reliability of the Raman method and its suitability to systematically analyze different cartilage structures and to decode their structural differences on the molecular scale.

### 2.5. FTIR and SR-FTIR Results

**Figure 8:**
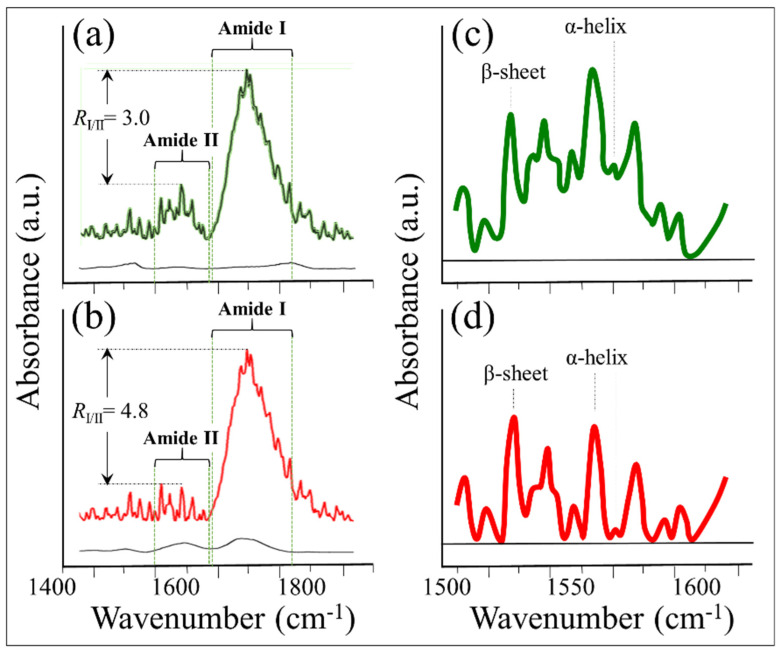
FTIR spectra collected in the spectral region 1400 to 1800 cm^−1^ for cartilage tissue grown on (**a**) NanoCliP-FD gel and (**b**) atelocollagen scaffolds. In (**c**,**d**), the enlarged spectral zone 1500–1600 cm^−1^ of the amide II vibrations is shown as collected on cartilage samples grown onto NanoCliP-FD gel and atelocollagen scaffolds, respectively.

In order to confirm the Raman results relating to higher α-helix hydration in the collagen structure of cartilage grown on the NanoCliP-FD gel scaffold as compared to atelocollagen, we performed FTIR characterizations of the two cartilage samples. Figure 8a,b show the FTIR spectra collected in the spectral region 1400~1800 cm^−1^ for cartilage tissue grown onto NanoCliP-FD gel and atelocollagen scaffolds, respectively. The selected spectral region includes amide I (mainly contributed by carbonyl C=O stretching [51]) and amide II (mainly contributed by N-H in-plane bending and C-N stretching [52]) vibrations. Differences in the FTIR amide I/amide II ratio have been associated with variations in protein secondary or tertiary conformations [53,54]. In particular, a lowered amide I/amide II ratio, *R*_I/II_, has been associated with a lower helicity level compared to pure collagen type II [55]. As seen from Figure 8, the cartilage grown on the NanoCliP-FD gel scaffold had a lower ratio than that grown on the atelocollagen one (*R*_I/II_ = 3.0 vs. 4.8; cf. labels in inset). Lowered helicity is indeed confirmation of helical hydration [56,57] and supports Raman analyses. Amide II bands have not yet been subjected to quantitative analyses because of their lower protein conformational sensitivity compared with amide I. However, the peaks at 1540 (with a shoulder at 1545) and 1551 cm^−1^ are usually assigned to an α-helical structure, while the peak at around 1521 cm^−1^ is assigned to β-sheet [57,58]. Figure 8c,d, which display the enlarged spectral zone 1500 to 1600 cm^−1^ of the amide II vibrations for cartilage samples grown onto NanoCliP-FD gel and atelocollagen scaffolds, respectively, suggest a higher fraction of α-helix in the former tissue, although in a more hydrated configuration.

**Figure 9:**
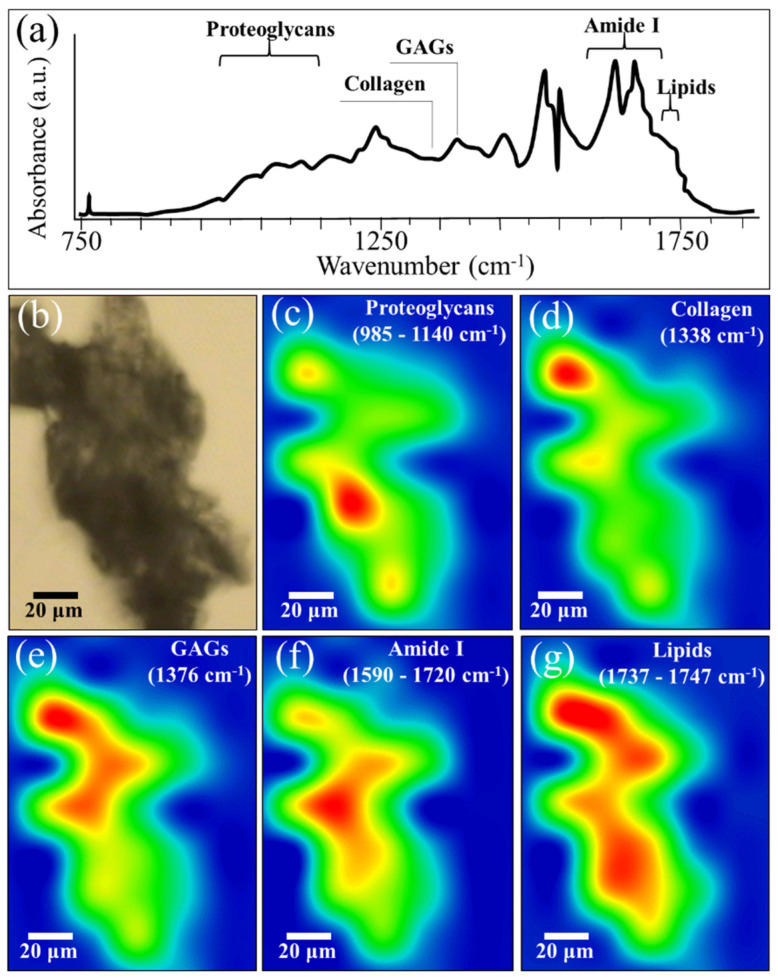
The spectrum in (**a**) shows the average SR-FTIR spectrum obtained on the cross-section of the cartilage sample grown onto the NanoCliP-FD gel scaffold given in the bright-field optical micrograph (**b**). In (**c**–**g**), related SR-FTIR images are given for proteoglycan, collagen, GAGs, amide I, and lipids, respectively (cf. mapping wavenumbers in inset to each figure).

Figure 9a shows the average SR-FTIR spectrum obtained on a cross section of the cartilage sample grown onto the NanoCliP-FD gel scaffold (location shown in the bright-field optical micrograph in Figure 9b. The SR-FTIR spectrum revealed several spectroscopic features in agreement with the FTIR characterization reported above in this section. Figure 9c–g represent the related SR-FTIR images for proteoglycan, collagen, GAGs, amide I, and lipids, respectively (cf. mapping wavenumbers in the inset to each figures). The so-called “collagen triplet” at 1203, 1234, and 1280 cm^−1^, often used to identify collagen in other tissues, is of limited utility in the analysis of cartilage, because it overlaps the sulphate peak at 1240 to 1245 cm^−1^ arising from sulphates [59,60], namely, the major components of GAGs in the cartilage matrix. For this reason, we preferred to map the weaker collagen band at 1338 cm^−1^. The SR-FTIR maps basically confirmed the morphological findings of the Raman results given in Section 2.3 by showing clear signals of proteoglycans and GAGs in the cartilage structure of the NanoCliP-FD scaffold. In addition to giving the spatial distributions of specific molecules along the scaffold cross-section, SR-FTIR mapping revealed the presence of large fraction of proteins (amide I band at 1590 to 1720 cm^−1^) and lipids (1737–1747 cm^−1^). An important feature of the SR-FTIR spectrum compared to the FTIR one resides in the clear distinctions of different sub-bands in the amide I region (cf. Figure 8a,b and Figure 9a). The prominent band at ~1635 cm^−1^ has been associated with living chondrocytes in cartilage explants and may be associated with matrix regeneration [61]. In the present study, chondrocytes in the NanoCliP-FD scaffold showed histological and spectroscopic evidence of hyaluronan expression (cf. Figure 3c). The band at 1635 cm^−1^ may also represent β-secondary structures within the globular heads of newly synthesized collagen produced by activated chondrocytes (i.e., both within the cells and in the matrix as precursors to fibril formation). Taken together, these results suggest that a strong signal at 1635 cm^−1^ marks chondrocytes’ activation in the cartilage [62]. In contrast, the equally strong signals seen at above 1660 cm^−1^ represented the amide I peak of the collagen helix [59] (cf. Figure 9a) in agreement with the histological evaluation of type II collagen expression within the cartilage matrix by means of immunostaining (cf. Figure 3d) [63]. In summary, SR-FTIR data confirmed that the NanoCliP-FD scaffold promoted the formation of high-quality cartilage.

## 3. Discussion

An important finding of the present study was the markedly different intensity of the S-S band located at 495 cm^−1^ in the Raman spectra of cartilage tissue grown on different scaffolds. This band was taken as a marker for TPI enzymatic catalysis. As already mentioned in Section 2.3, TPI is an extremely efficient metabolic enzyme that catalyzes the interconversion between DHAP and G3P in glycolysis and gluconeogenesis [31,32]. The Raman band at 495 cm^−1^, representative of TPI, gives a path to monitor in situ the efficiency of chondrocyte metabolism in cartilage formation and regeneration. Moreover, this spectroscopic finding also unfolded a so-far unreported mechanism of chondrocyte activation as exerted by the NanoCliP-FD gel scaffold, which we could label as “pullulan TPI uplift”.

TPI is one of the regulatory pathways governing the extracellular matrix (ECM) turnover by which the resident cells chondrocytes manage to build and maintain healthy cartilage. Proteomic analyses of the secretome have been published and extensively discussed in order to clarify the metabolic state of the cells and how chondrocytes actually operate [64]. Chondrocytes’ metabolism is strongly influenced by the ECM microenvironment; however, in a reciprocal interaction, the chondrocyte anabolic phenotype governs ECM composition by promoting growth factors and providing the morphogenetic proteins that regulate the organization and, ultimately, the quality of cartilage tissue [65,66]. Accordingly, the presence of a scaffold that positively promotes the activity of the anabolic chondrocyte phenotype could be key in accelerating cartilage regeneration. As shown by the present data, the NanoCliP-FD gel scaffold, which consists of a pullulan structure, namely, a long-chain polymeric structure of α-D-glucose rings (cf. Section 2.2), appears to fulfill this function.

**Figure 10:**
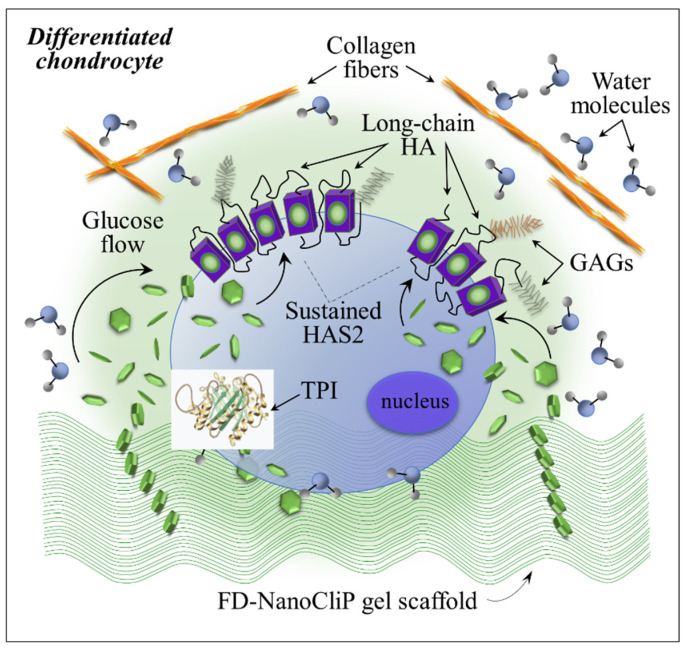
Schematic of metabolic effects of the pullulan molecules of the NanoCliP-FD gel scaffold on chondrocytes.

Chondrocytes build, remodel, and maintain the structure and functional integrity of cartilage ECM in the absence of vascular supply (i.e., in a relatively hypoxic environment); they consequently possess limited regenerative capacity under conditions of cellular stress. It is known that glucose is essential in sustaining chondrocyte metabolism, while also being a precursor for key ECM components [67]. Elevated glucose concentrations in the chondrocyte environment were found to promote ECM production, to sustain HAS2 gene expression, and to support hyaluronan synthase. This resulted in an increased glycolytic rate for differentiated chondrocytes. All of these characteristics were observed in the present in vitro experiments for the case of the NanoCliP-FD gel scaffold, thus showing the importance of the pullulan structure as a source of glucose and a fuel source for chondrocyte activation and cartilage formation. A schematic draft summarizing the metabolic effects induced by the pullulan molecules of the NanoCliP-FD gel scaffold on chondrocytes is given in Figure 10. In summary, elevated levels of glucose in ECM, as induced by the presence of pullulan, accelerated glucose uptake by differentiated chondrocytes and promoted HA-rich matrix production. The presently unfulfilled need for novel strategies in cartilage restoration calls for new approaches capable of satisfactory long-term results. Although further research is needed to fully characterize the impact of pullulan scaffolds on ECM structure and chondrocytes’ metabolism, the present data clearly show that the glucose eluted from the pullulan scaffold could exert beneficial effects in boosting TPI and HA formation, and consequently allowing the expedited production of high-quality cartilage.

## 4. Materials and Methods

### 4.1. Preparation of Fibronectin-Coated NanoCliP-FD Gel

A fibronectin-coated NanoCliP-FD gel was prepared according to the method reported by Hashimoto et al. [7] and Horiguchi et al. [10]. Using the procedure described in those reports, a coat of fibronectin was applied to the scaffolds to stimulate cellular adhesion. The NanoCliP-FD matrix was soaked in a fibronectin solution (Wako Laboratory Chemicals, Osaka, Japan) for 6 h. It was then rinsed twice in ethanol and dried. After the culture media containing cells was added to the resulting fibronectin-coated NanoCliP-FD matrix, aqueous media with cells was absorbed into the matrix and a NanoCliP-FD gel was yielded. The dimensions of the NanoCliP-FD gel and atelocollagen sponge were 10 × 1 × 1 mm and 3 × 3 × 2 mm, respectively.

### 4.2. Cell Culture

Human periodontal ligament-derived stem cells (PDLSCs) were purchased from ScienCell Research Laboratories, Inc. (Periodontal Ligament Fibroblasts, Human, Catalog #2630) (Carlsbad, CA, USA). The cells were cultured in Dulbecco’s minimum essential medium (DMEM; Nacalai Tesque, Kyoto, Japan) supplemented with 10% FBS, 100 U/mL penicillin, and 100 μg/mL streptomycin (Normal Medium) or Chondrogenic Differentiation Medium (PromoCell, Heidelberg, Germany) at 37 °C in an atmosphere containing 5% CO_2_. The PDLSCs were seeded in 20 μL-containing 24-well plates at a concentration of 5.0 × 10^6^/mL onto two types of scaffolds, namely, NanoCliP-FD gel and atelocollagen Honeycomb Sponge (Koken, Tokyo, Japan). After 2 h incubation in CO_2_, we added 1 mL of basic medium (DMEM) supplemented with 100 mM non-essential amino acids, 100 U/mL penicillin 100 μg/mL streptomycin, and 10% fetal bovine serum (FBS). After further culturing in a CO_2_ incubator for 24 h, the basic medium was replaced with a Chondrogenic Differentiation Medium (PromoCell, Heidelberg, Germany). The culture medium was changed every 2 to 3 days. To reduce cell loss, cell seeding onto scaffolds (10^4^ cells/20 μL/scaffold) was made by delivering cells in successive rates of small quantities. Cell culture experiments were repeated twice per each type of scaffold.

### 4.3. Histochemical Analyses by Safranin O and Picrosirius Red Staining

After cultivation for 2 weeks, the scaffolds were removed from the 24-well plate and set in 4% paraformaldehyde (Wako Pure Chemical Industries, Osaka, Japan). Next, safranin O staining was performed and the samples were decalcified with 20% ethylene diamine tetra-acetic acid and embedded in paraffin. The cartilage tissue was sliced in 10 µm-thick sagittal slices, and stained with Safranin O and Fast Green. Slices were obtained by microtome.

In performing the Picrosirius red staining, dewaxed and hydrated paraffin sections were used to wash the slides in distilled water. Then, the sections were stained in Picrosirius red solution (Muto Pure Chemicals Co., Ltd., Tokyo, Japan) for 20 min and washed in two changes of acidified water. The stained sections were treated to physically remove most of the water from the slides by vigorous shaking and dehydrated in three changes of isopropyl alcohol. After staining, the slices were analyzed with a BZ-X710 fluorescence microscope (Keyence, Osaka, Japan).

### 4.4. Immunohistochemical Analysis for Hyaluronan and Collagen Type 2

Because Versican G 1 domain binds specifically to hyaluronan and does not bind to other glycosaminoglycans, it can be used for hyaluronan detection [68,69]. Sections were stained using a biotinylated hyaluronic acid binding protein probe [70]. Tissue sections were then treated with 0.3% hydrogen peroxide in methanol at room temperature for 15 min for blocking. Biotinylated hyaluronic acid binding protein Versican G1 (1:50; Hokudo, Hokkaido, Japan) in phosphate-buffered saline (PBS) was then added, and the slides were kept in a humid chamber at room temperature for 15 min. The tissue sections were then incubated for 10 min with peroxidase-conjugated streptavidin (NICHIREI CORPORATION, Tokyo, Japan, 1:833 dilution). The sites of peroxidase activity were determined using diaminobenzidine (DAB; DAKO EnVision™ detection system, Agilent, Santa Clara, CA, USA) as the substrate. Counter staining was performed with Mayer’s hematoxylin.

Immunohistochemistry staining for collagen type II was performed according to the method given by Shibata et al. [71]. Deparaffinizated specimens were digested with testicular hyaluronidase in PBS (Sigma) at 37 °C for 3 h and washed in PBS-T (PBS containing 0.05% Tween 20) three times for 5 min each. They were then stained with rabbit polyclonal antibody anti type II collagen (Cosmo Bio Co., Ltd., Tokyo, Japan) (1:300~500 in 1% BSA in PBS) at 4 °C overnight. The slices were treated with biotin-labeled anti-rabbit IgG (HISTOFINE SAB kit, Nichirei Biosciences Inc., Tokyo, Japan), at room temperature for 15 min, and washed three times in PBS-T. Next, sections were treated with peroxidase-labeled streptavidin (from the HISTOFINE SAB kit), at room temperature for 10 min and washed three times PBS-T. Finally, slices were treated with DAB (from the HISTOFINE SAB kit) to reveal any reaction. Lastly, we confirmed that there was no nonspecific staining and analyzed the slices using the BZ-X710 fluorescence microscope.

### 4.5. Enzyme-Linked Immunosorbent Assay (ELISA) Kit

The culture supernatants were collected after 2 weeks and measured by ELISA. Concentrations of melanoma inhibiting activity (MIA) and hyaluronan in culture supernatants were measured by ELISA using a human MIA and Hyaluronan Assay kit (R&D Systems, Minneapolis, MN, USA). The MIA in tissue culture supernatant measured by quantitative ELISA can be used as a marker for differentiated chondrocytes [72].

### 4.6. Raman Analyses

In situ Raman spectra were collected using a highly sensitive instrument (LabRAM HR800, Horiba/Jobin-Yvon, Kyoto, Japan) with a 20× optical lens. The spectroscope operated in microscopic measurement mode with confocal imaging in two dimensions. A holographic notch filter within the optical circuit was used to efficiently achieve high-resolution spectral acquisitions. A spectral resolution of 1.5 cm^−1^ was obtained using a 532 nm excitation source operating at 10 mW. The Raman scattered light was monitored by means of a single monochromator connected to an air-cooled charge-coupled device detector (Andor DV420-OE322; 1024 × 256 pixel). The acquisition time was fixed at 10 s. Thirty spectra were collected at different locations on each sample and averaged at each analysis time-point. Raman spectra were deconvoluted into Gaussian–Lorentzian sub-bands using commercially available software (Origin 9.1, OriginLab Co., Northampton, MA, USA).

### 4.7. Raman Imaging

Raman maps of the cartilage tissue grown on different scaffolds were obtained by means of a dedicated instrument (RAMANtouch, Nanophoton Co., Osaka, Japan). This instrument was operated in microscopic measurement mode using the two-dimensional confocal imaging mode. A grating of 300 g/mm was used, and the source of excitation was at 532 nm. The spectral resolution in Raman mapping was 1.2 cm^−1^ (spectral pixel resolution equaled 0.3 cm^−1^/pixel), and an objective lens with 10× magnification with a numerical aperture of 0.3 was applied to the microprobe. We fixed the excitation laser power at 0.36 mW at the source. The Raman spectra that we acquired were deconvoluted into Gaussian–Lorentzian sub-bands. We performed this deconvolution with commercially available software (Origin 9.1). We constructed the Raman maps with commercially available software (Raman Viewer, Nanophoton Co., Osaka, Japan).

### 4.8. Fourier Transform Infrared Spectroscopy (FTIR) Analysis

Infrared spectra were acquired using a FTIR spectrophotometer (FT-720; Horiba, Ltd., Kyoto, Japan). The spectral resolution of this equipment was 1 cm^−1^ (over the range of 400–4000 cm^−1^). Spectral acquisition and pre-processing of raw data, which included baseline subtraction, smoothing, normalization, and fitting of the raw spectra, were carried out using commercially available software (Origin 9.1).

### 4.9. Synchrotron Radiation-Based FTIR

The cartilage tissue on the scaffolds was evaluated by synchrotron radiation-based (SR) FTIR. To characterize the content and distribution of collagen and proteoglycan in the cartilage tissue, SR-FTIR spectromicroscopy was performed with the BL15 beam line at the Ritsumeikan University SR Center (Kusatsu-shi, Shiga-ken, Japan). Sample preparation and SR-FTIR spectral analysis were performed according to a previously described procedure [11,73]. Briefly, paraffin-embedded scaffolds were sagittally sectioned into 5 μm slices, which were placed onto BaF_2_ substrates (Pier Optics Co., Ltd., Gunma, Japan) immediately after slicing. Sections were then dried overnight under vacuum following dewaxing and dehydration. Spectra were recorded using a Nicolet™ Continuμm™ (Thermo Fisher Scientific, Waltham, MA, USA) equipped with a 250 × 250 μm^2^ liquid nitrogen-cooled MCT/A detector, a 32X/NA0.65 Schwarzschild objective, a motorized knife-edge aperture, and a Prior XYZ motorized stage coupled with a Nicolet 6700 spectrometer (ThermoFisher, Waltham, MA, USA) equipped with a Michelson interferometer. Spectral and spatial resolutions were 0.4 cm^− 1^ and 10 μm, respectively. The distribution of collagen and proteoglycan was mapped using the amide I signal (at 1624–1675 cm^− 1^); signals representative for lipids (at 1737–1747 cm^− 1^), glycosaminoglycan (GAGs) (at 1376 cm^−1^), collagen type II (at 1338 cm^−1^), and proteoglycan (at 984–1140 cm^− 1^), were also used for imaging [74]. 

### 4.10. Statistical Analysis

Data were tested for their statistical significance according to the Student’s *t* test using GraphPad Prism version 6.04 for Windows (GraphPad Software, La Jolla, CA, USA).

## 5. Conclusions

Colonization of chondrocytes and the subsequent formation of cartilage tissue in vitro was analyzed in the case of PDLSCs cultured onto NanoCliP-FD gel and atelocollagen scaffolds. Immunohistochemical analyses and spectroscopic characterizations performed by Raman, FTIR, and SR-FTIR consistently showed that cartilage tissue formed on both types of scaffolds. However, the NanoCliP-FD gel scaffold promoted the formation of cartilage tissue at a speed ~5 times faster compared to the atelocollagen one under the same culture conditions.

Raman spectroscopic analyses and imaging revealed several important physiological circumstances. The relative intensity of the peculiar S-S stretching band of TPI, an enzyme essential in the glycolytic pathway and cartilage metabolism, and a measure of the functionality of chondrocytes in culture, was significantly higher in cartilage grown onto the NanoCliP-FD gel scaffold compared to the atelocollagen one. Moreover, the NanoCliP-FD gel scaffold greatly promoted the formation of HA and supported a more ordered and hydrated protein structure. FTIR and SR-FTIR basically confirmed these findings.

In conclusion, this study demonstrates that a fast synthesis of high-quality cartilage is possible using NanoCliP-FD gel scaffolds, thus opening a clinical path for their application in maxillofacial surgery of the nose, ears, and articular disk of the temporomandibular joint. The novelty of a non-destructive Raman method, capable of evaluating in situ the matrix quality while also revealing the cells’ metabolic response, is also emphasized.

## Figures and Tables

**Table 1:** Wavenumbers and vibrational assignments of the main Raman bands found in the spectrum of the NanoCliP-FD gel scaffold (band labels correspond to those shown in Figure 4a.

Band Label	Wavenumber (cm^−1^)	Assignment	Ref.
1	234	Glucose C-O torsion	[15]
2	276	Glucose C-O torsion	[15]
3	359	Glucose C-C-O bending	[15]
4	429	Glucose ring C-C-O bending	[15]
5	534	Glucose C-C and C-O stretching	[16]
6	580	Glucose C-O stretching	[15]
7	843	Glucose anomeric C-H bending	[17]
8	921	C-C-O bending	[17]
9	1060	Glucose C-C-H bending	[17]
10	1125	C-O-C stretching in ester	[18]
11	1141	Glucose C-O stretching	[18]
12	1234	Amide III	[18]
13	1278	CH2 deformation	[18]
14	1455	C-H and C-H2 bending	[18]
15	1476	C-H and C-H2 bending	[18]

**Table 2:** Wavenumbers and vibrational assignments of the main Raman bands found in the spectrum of the atelocollagen scaffold (band labels correspond to those shown in Figure 4b.

Band Label	Wavenumber (cm^−1^)	Assignment	Ref.
1	307	Pro-Pro-Gly (tripeptide)	[7,19,20,21,22]
2	402	Pro-Pro-Gly (tripeptide)	[7,19,20,21,22]
3	561	Amide VI (-CO-NH-)	[19]
4	763	Amide VI (-CO-NH-)	[19]
5	816	C-C backbone	[19]
6	858	C-C (Pro)	[7]
7	874	C-C (Pro)	[7]
8	921	C-C (Pro)	[7]
9	938	C-C α-helix	[7]
10	1004	C-C stretch (Phe)	[7]
11	1034	C-OH bending	[21]
12	1106	C-H2 wag (glutamic acid)	[20]
13	1177	C-C-H bending (Tyr)	[22]
14	1247	Amide III	[21]
15	1268	Amide III	[21]
16	1324	Amide III	[21]
17	1420	COO- stretching	[7]
18	1455	CH2, CH3 bending	[22]
19	1655	Amide I	[20]

## Data Availability

Not applicable.
